# Immunoreactivity of valosin-containing protein in sporadic amyotrophic lateral sclerosis and in a case of its novel mutant

**DOI:** 10.1186/s40478-014-0172-0

**Published:** 2014-12-10

**Authors:** Takashi Ayaki, Hidefumi Ito, Hiroko Fukushima, Takeshi Inoue, Takayuki Kondo, Akito Ikemoto, Takeshi Asano, Akemi Shodai, Takuji Fujita, Satoshi Fukui, Hiroyuki Morino, Satoshi Nakano, Hirofumi Kusaka, Hirofumi Yamashita, Masafumi Ihara, Riki Matsumoto, Jun Kawamata, Makoto Urushitani, Hideshi Kawakami, Ryosuke Takahashi

**Affiliations:** Department of Neurology, Wakayama Medical University, 811-1, Kimiidera, Wakayama, 641-8510 Japan; Department of Pathology, Osaka City General Hospital, 2-13-22, Miyakojima-Hondori, Miyakojima-ku, Osaka, 534-0021 Japan; Center for iPS Cell Research and Application, Kyoto University, 53, Kawahara-cho, Shogoin, Sakyo-ku, Kyoto, 606-8507 Japan; Department of Neurology, Kyoto University Graduate School of Medicine, 53, Kawahara-cho, Shogoin, Sakyo-ku, Kyoto, 606-8507 Japan; Kyoto Municipal Rehabilitation Center for Physically Disabled People, 30, Mibusennen-cho, Nakagyo-ku,, Kyoto, 604-8854 Japan; Takumi Medical Corporation, Neurology Clinic, 7-8, Takarayama-cho, Toyonaka, Osaka, 561-0893 Japan; Tazuke Kofukai Foundation, Medical Research Institute and Kitano Hospital, 2-4-20, Ohgimachi, Kita-ku, Osaka, 530-8480 Japan; Department of Epidemiology, Research Institute for Radiation Biology and Medicine, Hiroshima University, 1-2-3, Kasumi, Minami-ku, Hiroshima, 734-8553 Japan; Department of Neurology, Osaka City General Hospital, 2-13-22, Miyakojima-Hondori, Miyakojima-ku, Osaka, 534-0021 Japan; Department of Neurology, Kansai Medical University, 2-5-1, Shin-machi, Hirakata, Osaka, 573-1010 Japan; Department of Stroke and Cerebrovascular Diseases, National Cerebral and Cardiovascular Center, 5-7-1, Fujishirodai, Suita, Osaka, 565-8565 Japan; Department of Epilepsy, Movement Disorders and Physiology, Kyoto University Graduate School of Medicine, 53, Kawahara-cho, Shogoin, Sakyo-ku, Kyoto, 606-8507 Japan; Department of Neurology, School of Medicine, Sapporo Medical University, South 1 West 17, Chuo-ku, Sapporo, Hokkaido 060-8556 Japan

**Keywords:** Amyotrophic lateral sclerosis, Paget disease of bone, Valosin-containing protein (VCP), Inclusion body myopathy with early-onset Paget disease and frontotemporal dementia (IBMPFD), TAR DNA-binding protein 43 kDa (TDP-43), Golgi apparatus fragmentation

## Abstract

**Background:**

Mutations in the *valosin-containing protein (VCP)* gene were first found to cause inclusion- body myopathy with early-onset Paget disease and frontotemporal dementia (IBMPFD). Mutations in the *VCP* gene were later reported to occur in familial amyotrophic lateral sclerosis (ALS). But the role of VCP in the neurodegenerative processes that occur in ALS remains unknown. The purpose of the present study was to elucidate the role of VCP in the neurodegeneration seen in sporadic and *VCP* mutant ALS.

**Results:**

Immunohistochemistry demonstrated that the frequency of distinct VCP-positive nuclei of spinal motor neurons of patients with sporadic ALS (SALS) and the ALS with *VCP* novel mutation (ALS-VCP, M158V) was increased, compared with that of the control cases. No VCP-positive inclusion bodies were observed in SALS patients, a ALS-VCP patient or in control subjects. Neuropathologic examination of the ALS-VCP case showed loss of motor neurons, the presence of Bunina bodies, and degeneration of the corticospinal tracts. Bunina bodies detected in this case were confirmed to show immunohistochemical and ultrastructural features similar to those previously described. Furthermore, neuronal intracytoplasmic inclusions immunopositive for TAR DNA-binding protein 43 kDa (TDP-43), phosphorylated TDP-43, ubiquitin (Ub), p62, and optineurin were identified in the spinal and medullary motoneurons, but not in the neocortex. Gene analysis of this ALS-VCP patient confirmed the *de novo* mutation of M158V, which was not found in control cases; and bioinformatics using several *in silico* analyses showed possible damage to the structure of VCP. Immunocytochemical study of cultured cells showed increased cytoplasmic translocation of TDP-43 in cells transfected with several mutant *VCP* including our patient’s compared with wild-type VCP.

**Conclusion:**

These findings support the idea that VCP is associated with the pathomechanism of SALS and familial ALS with a *VCP* mutation, presumably acting through a dominant-negative mechanism.

**Electronic supplementary material:**

The online version of this article (doi:10.1186/s40478-014-0172-0) contains supplementary material, which is available to authorized users.

## Introduction

Valosin-containing protein (VCP) is a ubiquitous member of the AAA-ATPase supergene family. VCP is known to play an important role in cellular activities including ubiquitin (Ub) -dependent protein degradation [1], chromatin-associated protein degradation [2], messenger ribonucleic acid (mRNA) metabolism [3], autophagy [4], anti-apoptotic function [5], and post-mitotic Golgi apparatus reassembly [6]. Mutations in the *VCP* gene were first found to cause inclusion-body myopathy with early-onset Paget disease and frontotemporal dementia (IBMPFD) [7,8]. IBMPFD is an autosomal dominantly inherited disorder with variable penetrance of 3 predominant phenotypic features, i.e., myopathy, Paget disease of bone, and frontotemporal dementia (FTD) [9]. The penetrance of the gene is 82% for myopathy, 49% for Paget’s disease, and 30% for FTD [10]. IBMPFD patients with *VCP* mutations can develop disorders in other organ systems including sphincters [11], cardiac muscles [12], auditory system [13], and liver [14], as well as in visuoconstructive ability [14]. Neurodegenerative diseases associated with a *VCP* mutation encompass scapuloperoneal muscular dystrophy and dropped head syndrome [15], Parkinson’s disease [16-18], hereditary spastic paraplegia [19], and cerebellar ataxia [20]. In addition to mutations in *VCP* [7,8], mutations in *Heterogeneous Nuclear Ribonucleoprotein A2B1* (*HNRNPA2B1*) and *Heterogeneous Nuclear Ribonucleoprotein A1* (*HNRNPA1*) [21] have been identified in families with IBMPFD. Given these observations, the name of multisystem proteinopathy (MSP) has been proposed, using the nomenclature of MSP1 for IBMPFD caused by a *VCP* mutation, MSP2 for IBMPFD related to an *HNRNPA2B1* mutation, MSP3 for IBMPFD related to an *HNRNPA1* mutation, and MSP4 for IBMPFD due to some unidentified gene [22]. Clinically, 37.7% patients of IBMPFD with a *VCP* mutation (MSP1) develop FTD [9]. FTD cases with a *VCP* mutation (MSP1) present with TAR DNA-binding protein 43 kDa (TDP-43) and ubiquitin-positive short dystrophic neurites and frequently lentiform neuronal intranuclear inclusions in their neocortex [23-25]. On the other hand, only rare VCP-positive neuronal intranuclear inclusions are detected, and those that are detected lack the characteristic lentiform morphology [23]. This finding suggests that TDP-43 and ubiquitin positive-inclusions do not contain VCP and supports the idea that *VCP* gene mutations in IBMPFD produce a dominant-negative loss of VCP function [23,25].

Mutations in the *VCP* gene were later reported to occur in familial amyotrophic lateral sclerosis (ALS) [26]. A recent study with a large data set of patients with *VCP* mutations showed that 8.9% of these patients developed ALS [27]. Features of ALS with *VCP* mutations (ALS-VCP) are similar to those of sporadic ALS (SALS), including bulbar signs, spasticity, hyperreflexia, fasciculations, and electrophysiological evidence of lower motor neuron involvement such as denervation and reinnervation [27]. The neuropathology of ALS-VCP has been briefly described in 2 cases to date [26,28]. Both reports described TDP-43-positive intracytoplasmic inclusions in the motor neurons. These facts suggest that the neuropathologic features of ALS-VCP could be similar to those of SALS and that SALS would share its pathogenic role with ALS-VCP cases through dysfunction of VCP. To confirm this hypothesis, it is of importance to elucidate detailed pathological features of ALS-VCP and to compare them with those of SALS. However, specific inclusion pathology including their immunohistochemical properties and distributions, as well as detailed cytopathology of motor and non-motor neurons and glial cells, remain to be explored.

VCP immunoreactivity has been observed in Lewy bodies in Parkinson’s disease and dementia with Lewy bodies, in neuronal nuclear inclusions in polyglutamine diseases and intranuclear inclusion body disease, in Marinesco bodies [29], and in epidermal cells from patients with SALS [30]. VCP-positive nuclei have also been reported in neocortical neurons [31] and in muscle cells [20] from IBMPFD cases with a *VCP* mutation (MSP1). However, the distribution of VCP in ALS-VCP cases is unknown. Moreover, it remains to be investigated whether a *VCP* mutation leads to VCP-positive inclusions in the ALS phenotype, and whether VCP-positive structures are present in the motor neurons of SALS patients.

To elucidate the role of VCP in neurodegenerative processes in ALS, in the present study we examined the neuropathology of a patient with ALS and Paget disease of bone with a novel *VCP* mutation, as well as the immunohistochemical localization of VCP in SALS cases and in the ALS-VCP patient.

## Materials and methods

### VCP immunohistochemistry in ALS

We investigated specimens from lumbar spinal cord, hippocampus, and the motor cortex from 9 patients with pathologically confirmed SALS (age range, 54–82 years; mean, 63.2 years; 6 men and 3 women), 8 control subjects (age range, 50–86 years; mean, 69.5 years; 6 men and 2 women), and 1 patient with ALS associated with a novel *VCP* mutation. The clinical profiles of all of these cases are summarized in Table 1. Among SALS cases, there was no familial case and no history of IBM, Paget disease or FTD.

**Table 1:** **Clinical findings of patients with amyotrophic lateral sclerosis and of control subjects**

**Case no.**	**Age at death (years)**	**Gender**	**Diagnosis**	**Postmortem delay (hours)**	**Duration of illness (months)**	**Percentage of VCP-positive nuclei**
Control	cases					
1	75	M	Chronic lymphocitic leukemia	2.5	NA	0
2	75	M	Cerebral infarction	1	NA	0
3	86	M	Cerebral infarction	5	NA	0
4	75	M	Meningitis	3	NA	1.7
5	83	F	Intracerebral hemorrhage	12	NA	4
6	52	M	Cerebral infarction	12	NA	8.9
7	50	F	Myotonic Dystrophy	10	NA	0
8	60	M	Myasthenia Gravis	2.5	NA	0
ALS cases						
9	54	F	SALS	3.5	11	26.3
10	82	M	SALS	1.5	14	0
11	62	M	SALS	1.5	14	0
12	56	F	SALS	22.5	20	0
13	65	M	SALS	1.5	23	8.3
14	63	M	SALS	1.5	23	15
15	64	M	SALS	3	24	17.6
16	65	M	SALS	16	24	94.1
17	58	F	SALS	3.5	35	47.1
18	41	M	VCP-ALS	2	48	7.7

*M* male, *F* female, *VCP* Valosin-Containing Protein, *SALS* sporadic amyotrophic lateral sclerosis, *VCP-ALS* ALS with M158V VCP mutation.

For VCP immunohistochemistry, formalin-fixed, paraffin-embedded 6-μm-thick sections were deparaffinized and immunostained for VCP. We applied 2 distinct primary antibodies for VCP(mouse monoclonal [Cat. No. ab11433], Abcam plc, Cambridge, UK; 1:500; and rabbit polyclonal [Cat. No. AP6920b], Abgent, San Diego, California, USA; 1:75). Bound antibodies were detected with the appropriate VECTASTAIN Elite ABC Kit (Vector Laboratories, Burlingame, California, USA). We assessed staining specificity by replacing the primary antibodies with an appropriate amount of phosphate-buffered saline solution containing 3% bovine serum albumin. No deposits of reaction products were seen in the sections thus treated. Procedures involving the use of human material were performed in accordance with ethical guidelines set by Kyoto University.

For quantitative study of VCP-positive nuclei of the motor neurons, we obtained 3 distinct sections from each case, taken at an interval of 30 μm, to avoid counting the same nucleus twice. All observations were made by 2 examiners who had no information regarding the clinical history and condition of the individual providing the spinal specimen. Only anterior horn cells (AHCs) with a distinct nucleus and nucleolus were counted. The frequency of VCP-positive motor neurons in the lumbar cord was compared between control patients and SALS patients by the nonparametric Mann–Whitney U test using Prism software (GraphPad, La Jolla, USA).

### Clinical features of the ALS patient with a *VCP* mutation

**Figure 1:**
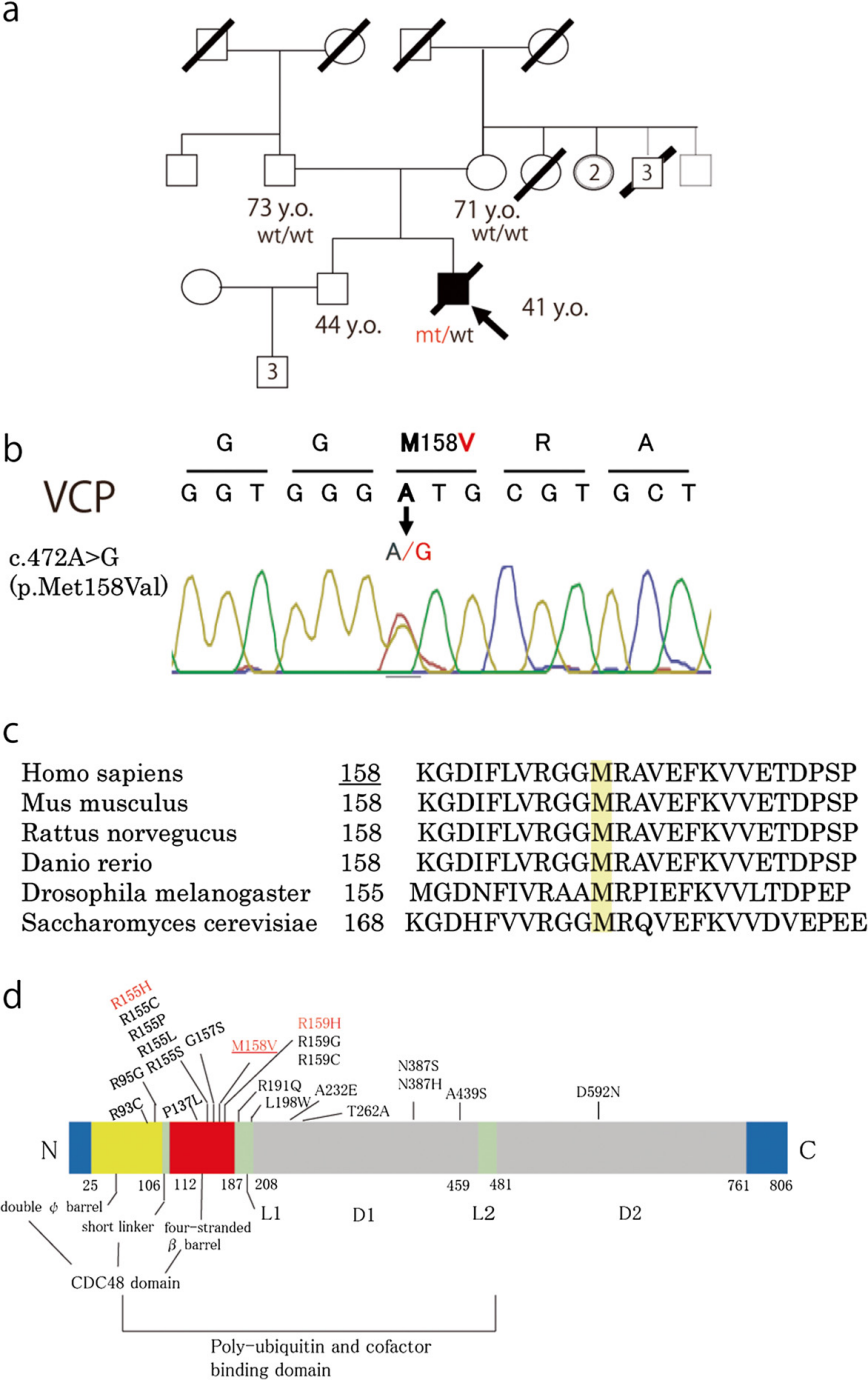
**Genetic information. (a)** The presented case had no family history of ALS, dementia, myopathy or Paget disease. Family members with diagonal black lines had already died. The ages indicated are those at the time of the proband’s death. **(b)** The proband carried the missense mutation of the *VCP* gene (c. 472A > G, p.M158V). Neither the father nor the mother carried the *VCP* mutation identified in the proband. **(c)** Sequence alignment for amino acids revealed the mutation site to be conserved across different species. **(d)** Domain structure of VCP protein and mutation site in this case and in reported mutations (Mutations with autopsy are shown in red type) associated with ALS and IBMPFD.

Our patient was a 41-year-old Japanese man without any family history of ALS, IBM, Paget disease or FTD (Figure 1a). He had developed right arm and leg weakness at age 36. Based on the results of elevated alkaline phosphatase up to 2,968 IU/l, bone radiographs, and electromyography, he was diagnosed at age 37 as having ALS and Paget disease of bone. He gradually developed weakness of arms and legs, dysarthria, dysphagia, and respiratory failure, which required percutaneous endoscopic gastrostomy and noninvasive positive-pressure ventilation at age 38. He was referred to our hospital at age 39. Examinations disclosed weakness in all extremities, muscle atrophy in the distal arms, and fasciculation in the right leg. Atrophy or fasciculation of the tongue was not evident. Patellar tendon reflexes were brisk, but Achilles tendon reflexes and those in the upper limbs were decreased. Neither pathologic reflexes nor clonus was observed in the extremities. Neuropsychological tests showed no evidence of FTD. A computed tomography (CT) scan showed systemic osteolytic change, but no atrophy of the brain. Needle electromyography (EMG) showed active and chronic denervation potential including spontaneous discharges and late recruitment in the left arm and leg. A frozen muscle specimen from the right quadriceps muscle at this time showed neurogenic changes without evidence of IBM. Base on the revised El Escorial criteria [32], the patient was diagnosed as clinically possible ALS.

His extremities gradually became weaker, resulting in a bedridden condition. Shortly after he developed a fracture of the right femoral bone, he died of respiratory failure at the age of 41. Throughout the clinical course he did not develop any cognitive decline or mood disorder, Parkinsonism, spastic paraplegia, cerebellar ataxia or decubitus ulcer. Also, the patient did not have any abnormalities in cardiac, hepatic, visual, auditory, sensory or autonomic systems. No frozen brain tissue was available.

### Genetic analysis

Genetic analysis of *VCP, Cu/Zn superoxide dismutase 1 (SOD1), TDP-43, fused in sarcoma (FUS), Charged multivesicular body protein 2b (CHMP2B), angiogenin, Sequestosome 1 (SQSTM1), and chromosome 9 open reading frame 72 (C9ORF72)* was approved by either the Kyoto University Graduate School of Medicine Ethical Committee or Hiroshima University Ethical Committee, and written informed consent was obtained from the above patient prior to his demise. DNA was extracted from a blood sample; and by using previously reported primers, we amplified the exons of each gene and sequenced them on anABI310 sequencer (Applied Biosystem, Foster City, California, USA). To detect *C9ORF72* expansion, we performed a repeat-primed polymerase chain reaction, as reported previously [33]. As a result we identified a novel heterozygous M158V mutation in the *VCP* gene (Figure 1b). This mutation was not present in the 1000 Genomes project (http://www.1000genomes.org/), dbSNP (http://www.ncbi.nlm.nih.gov/SNP/) or in the Human Genetic Variation Browser (includes genetic variations determined by exome sequencing of 1,208 Japanese individuals and genotyping data of common variations obtained from a cohort of 3,248 individuals [http://www.genome.med.kyoto-u.ac.jp/SnpDB/]). The other genes were normal. Conservation analysis of the mutated amino acid by using National Center for Biotechnology (NCBI) HomoloGene demonstrated conservation among species at the mutation site (Figure 1c). The mutation site in this case (amino acid 158 in VCP) was located near the reported mutation sites (amino acids 155 [26] and 159 [28] in VCP) within the same domain (Figure 1d). In Figure 1d, mutation sites in *VCP* gene associated with ALS and IBMPFD are also listed from the literature [8,9,13-15,26,28,31,34-39].

His parents were still alive and not affected with ALS or any other neurodegenerative diseases. His father was 73 years of age and had been diagnosed with prostate cancer, whereas his mother was 71 years old and healthy. Neither parent carried the *VCP* mutation identified in the proband. Haplotype analysis using 15 short tandem repeat markers supported their genetic kinship, confirming that the mutation occurred *de novo* in the present case. No sample from the patient’s brother was available.

### Neuropathologic examinations

For neuropathologic examinations, formalin-fixed, paraffin-embedded 6-μm-thick sections were deparaffinized and stained with hematoxylin and eosin (H&E) or used for Klüver-Barrera (KB) staining. For immunohistochemistry, after antigen retrieval by heat/autoclaving (10 min at 121°C in 10 mM anhydrous citric acid buffer, pH 6.0), the sections were immunostained as described above.

The antigens recognized by the primary antibodies used in this study were the following: TDP-43(rabbit polyclonal [Cat No. 10782-2-AP], ProteinTec Group Inc., Chicago, Illinois, USA; 1:200), phosphorylated TDP-43 (mouse monoclonal [Cat No. TIP-PDT-M01], Cosmo Bio Co., Ltd., Tokyo, Japan; 1:2000), trans-Golgi-network (sheep polyclonal [Cat No. 610832], Novus Biologicals, Inc, Littleton, Colorado, USA; 1:250), VCP(mouse monoclonal [Cat No. ab11433], Abcam plc, Cambridge, UK; 1:500; rabbit polyclonal [Cat No. AP6920b], Abgent, San Diego, California, USA; 1:75), FUS(rabbit polyclonal [Cat No. HPA008784], Sigma-Aldrich Inc., St. Louis, Missouri, USA; 1:100), CD68 (mouse monoclonal [Cat No. M0814], DAKO, Glostrup, Denmark; 1:100), p62(mouse monoclonal [Cat No. 610832], Becton Dickinson and Company, Franklin Lakes, New Jersey, USA; 1:700), ubiquitin(rabbit polyclonal [Cat No. U5379], Sigma-Aldrich Inc., St. Louis, Missouri, USA; 1:100), and optineurin (rabbit polyclonal [Cat No. 100000], Cayman Chemical Company, Ann Arbor, Michigan, USA; 1:200).

### Electron microscopy

In the case with the *VCP* mutation, the sample from formalin-fixed medulla was fixed in 2% glutaraldehyde with phosphate buffer (pH 7.4). After fixation, sections were cut into pieces about 1 mm thick, postfixed with 1% osmium tetroxide for 2 h, dehydrated, and embedded in epoxy resin. Each block was then cut into semithin sections about 1 μm in thickness and stained with toluidine blue. Appropriate regions were subsequently cut into ultrathin sections and stained with uranyl acetate and lead citrate for electron microscopy.

### Proteomics analysis

The effect of the newly detected missense mutation (M158V) and previously reported mutations of 2 autopsied ALS-VCP cases (R155H and R159H) was analyzed with Mutation Taster (http://www.mutationtaster.org), Sorting Intolerant From Tolerant (SIFT, http://sift.bii.a-star.edu.sg/), and PolyPhen-2 (http://genetics.bwh.harvard.edu/pph/).

### Vectors for *in vitro* analysis

To assess the functional importance of the novel mutation identified in this study, we created vectors (pcDNA3) expressing wild-type (WT) *VCP* and mutant *VCPs* (R155H, M158V, R159H and A232E) tagged with FLAG at their N-terminus.

### Cultured cells

SH-SY5Y and human embryonic kidney 293 T (HEK293T) cells were grown in Dulbecco’s Modified Eagle Medium (DMEM) supplemented with 10% fetal bovine serum, 1% non-essential amino acids, and 1% penicillin-streptomycin-L glutamine sodium.

### Plasmids

To generate FLAG-tagged VCP, we obtained WT VCP complementary deoxyribonucleic acid (cDNA) from a cDNA library and generated sequence variants, including R155H (464G > A), M158V (472A > G), R159H (476 G > A) and A232E (695C > A). WT and mutant VCPs were generated by PCR with primer pairs used to change the nucleotides and then inserted into the Not I/BamH I cloning site of the pcDNA3 vector. All constructs were verified by using the 3130xl Genetic Analyzer (Life Technologies, Carlsbad, California, USA. Cultured cells were transfected with the vector by using a FuGene HD transfection kit (Roche, Basel, Switzerland) according to the manufacturer’s protocol.

### Immunoblot analysis

Cells were lysed in lysis buffer containing 10 mM Tris–HCl (pH 7.6), 150 mM NaCl, 1% Triton X-100, 1% sodium deoxycholate (Na-DOC), 0.1% sodium dodecyl sulfate (SDS), and a protease inhibitor cocktail (Complete EDTA-free protease inhibitor; Nacalai Tesque). Nuclei and membrane fractions were removed by centrifugation. Lysates were separated by SDS-poly-acrylamide gel electrophoresis (SDS-PAGE), and proteins were then transferred to a polyvinylidene difluoride (PVDF) membrane. The membrane was incubated with the appropriate primary antibody, followed by incubation with horseradish peroxidase (HRP)-conjugated anti-rabbit or anti-mouse IgG (Santa Cruz Biotechnology Inc, Dallas, Texas, USA) secondary antibody. Immunoreactive proteins were visualized by using the Pierce ECL Western Blotting Substrate (Thermo Scientific Pierce, Kanagawa, Japan) and an LAS3000 scanning system (Fuji Film, Tokyo, Japan). The following primary antibodies were used in the immunoblot analysis: anti-VCP (rabbit polyclonal [Cat No. AP6920b], Abgent, San Diego, California, USA; 1:200) and anti-β-actin (mouse monoclonal [Cat No. A5441], Sigma-Aldrich Inc., St. Louis, Missouri, USA; 1:2,000).

### Immunocytochemistry

SH-SY5Y and HEK293T cells transfected with FLAG-conjugated WT or mutant *VCP* were fixed and immunostained with anti-TDP-43 or anti-FLAG, and stained with DAPI, at 48 h post-transfection. For immunofluorescence analysis of cell cultures, cultured cells were fixed with 4% PFA for 20 min, washed 3 times with PBS (2 min each), and then rendered permeable and blocked with 0.2% Triton X-100/5% goat serum in PBS for 15 min. The transfected cells were incubated with primary antibodies (anti-TDP-43 antibody, rabbit polyclonal, ProteinTech, 1:1000 and anti-FLAG M2, mouse monoclonal, Sigma-Aldrich Inc., St. Louis, Missouri, USA; 1:500, diluted in PBS containing 0.2% Triton X-100/5% goat serum) overnight and washed 3 times with PBS (5 min each). After the final wash, the cells were incubated with secondary antibodies (Alexa Fluor 488 donkey anti-rabbit IgG (H + L) or Alexa Fluor 546 donkey anti-mouse IgG (H + L), diluted in PBS containing 0.2% Triton X-100/5% goat serum) for 1 h, washed 3 times with PBS (5 min each), and mounted with Vectashield plus DAPI (Vector Laboratories, Burlingame, California, USA). Digital imaging was performed with an OLYMPUS FV-100 IX microscope (Olympus, Tokyo, Japan) using FV-10-ASW 3.1 software (Olympus). A blinded examiner counted more than 100 FLAG-tagged VCP-expressing cells from separate cultures for the presence of cytoplasmic TDP-43-positive cells. Results were analyzed by One-way ANOVA of Bonferroni’s multiple-comparison test using Prism software (GraphPad, La Jolla, USA).

## Results

### Immunohistochemistry for VCP in control subjects, SALS patients, and the *VCP* mutant case

Two distinct anti-VCP antibodies applied in the present study gave the same results.

**Figure 2:**
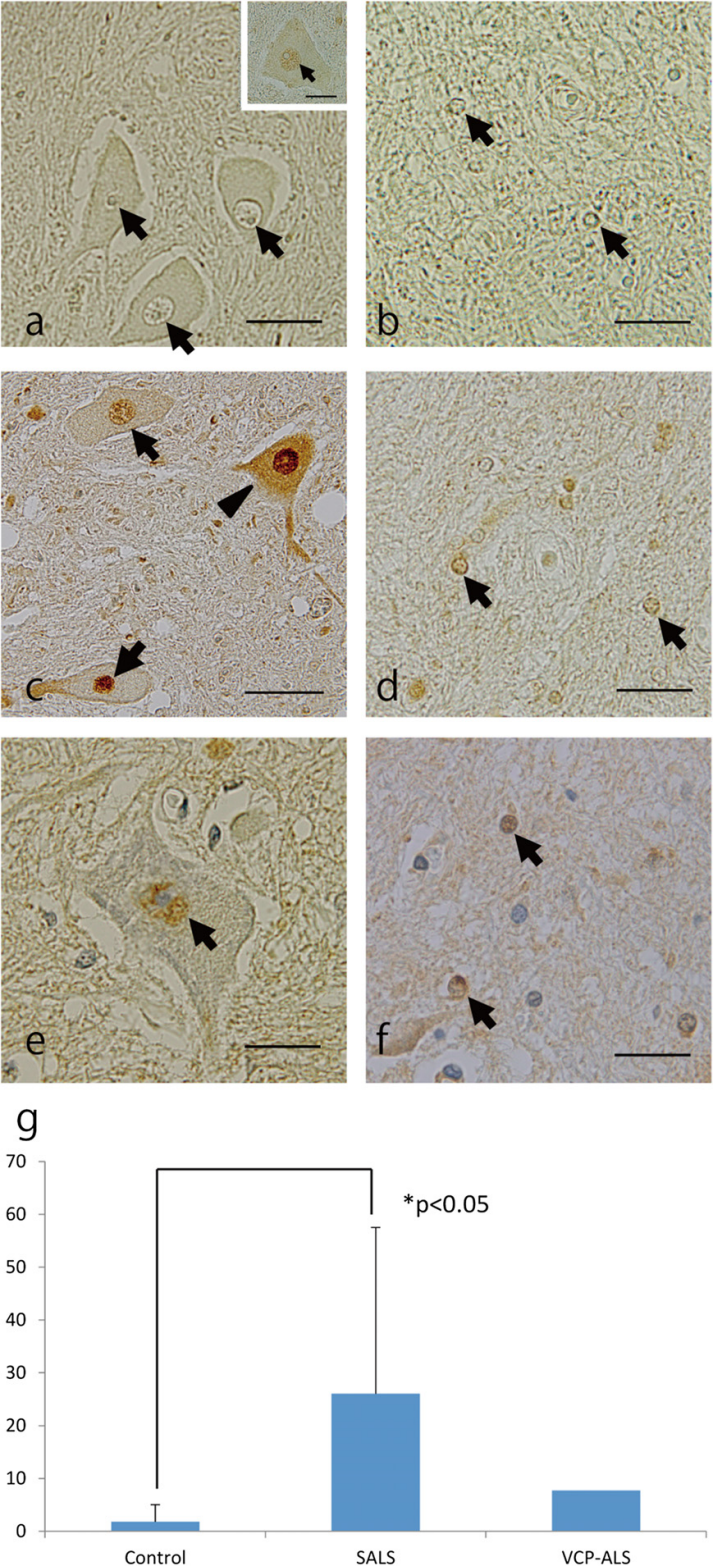
**Immunohistochemistry of VCP in the spinal cords. (a,b)** In the control case, neuronal (**a**, *arrows*) and glial (**b,** *arrows*) nuclei are devoid of immunoreactivity. The nucleus of this AHC (**a**, inset, arrow) in the control case was stained faintly with the anti-VCP antibody. **(c,d)** In the ALS cases the same antibody distinctly immunolabels nuclei of the AHCs (**c**, *arrows*) and those of the glial cells (**d,** *arrows*). The cytoplasm of the indicated AHC (**c**, *arrowhead*) is also stained diffusely. **(e,f)** In the ALS-VCP patient a nucleus of an AHC (**e**, *arrow*) and glial nuclei (**f**, *arrows*) give positive staining with the anti-VCP antibody. **(g)** The frequency of VCP-positive neuronal nuclei in the lumbar cord of the patients with SALS (mean ± standard deviation, 26.1 ± 31.5%) was significantly higher than that of the controls (1.8 ± 3.2%). Values are the means of the percentage of VCP-positive neuronal nuclei in each case; and error bars represent the standard deviation (*p < 0.05, Mann–Whitney U test). Immunohistochemistry was performed with the rabbit polyclonal antibody against VCP. *Scale bars* = 25 μm.

In the 8 controls, VCP-positive nuclei or cytoplasm was rarely observed in the AHCs investigated (Figure 2a); only a few nuclei of AHCs showed faint immunoreactivity against VCP (Figure 2a inset). Glial nuclei were virtually not stained in these subjects (Figure 2b). In contrast, AHCs from 9 SALS cases frequently showed positive VCP immunoreactivity in the cytoplasm and in the nucleus (Figure 2c). The staining intensity of neuronal nuclei of the SALS cases was much greater than that in the control subjects. Within the cytoplasm, the deposits of immunoreaction product were diffusely distributed, but this antibody recognized no intranuclear inclusion, skein-like inclusion, round hyaline inclusion or Bunina body. Moreover, the glial nuclei were also stained in the cases with SALS (Figure 2d). However, VCP-positive glial cytoplasmic inclusions (GCIs) were not observed.

We also identified some neuronal (Figure 2e) and glial (Figure 2f) nuclei that were immunoreactive for VCP in the ALS case with the heterozygous M158V *VCP* mutation (case 18 in the Table). In the ALS-VCP case, VCP immunoreactivity in the AHCs was less robust than that in the SALS cases. No VCP-positive inclusions were detected in any of the cases investigated.

In the motor cortex and hippocampus, there was no significant immunoreactivity for VCP except for faint immunoreactivity of glial nuclei.

The frequency of VCP-positive nuclei in the lumbar cord of patients with SALS (mean ± standard deviation, 26.1 ± 31.5) was significantly higher (p < 0.05, Mann–Whitney U test) than that for the control subjects (1.8 ± 3.2%; Figure 2g). In the ALS-VCP case 7.7% of the AHC nuclei were VCP positive. On serial section analysis, there was no apparent relationship between VCP immunoreactivity and intracytoplasmic TDP-43 accumulation.

### Neuropathologic examinations of the patient with M158V ALS-VCP

**Figure 3:**
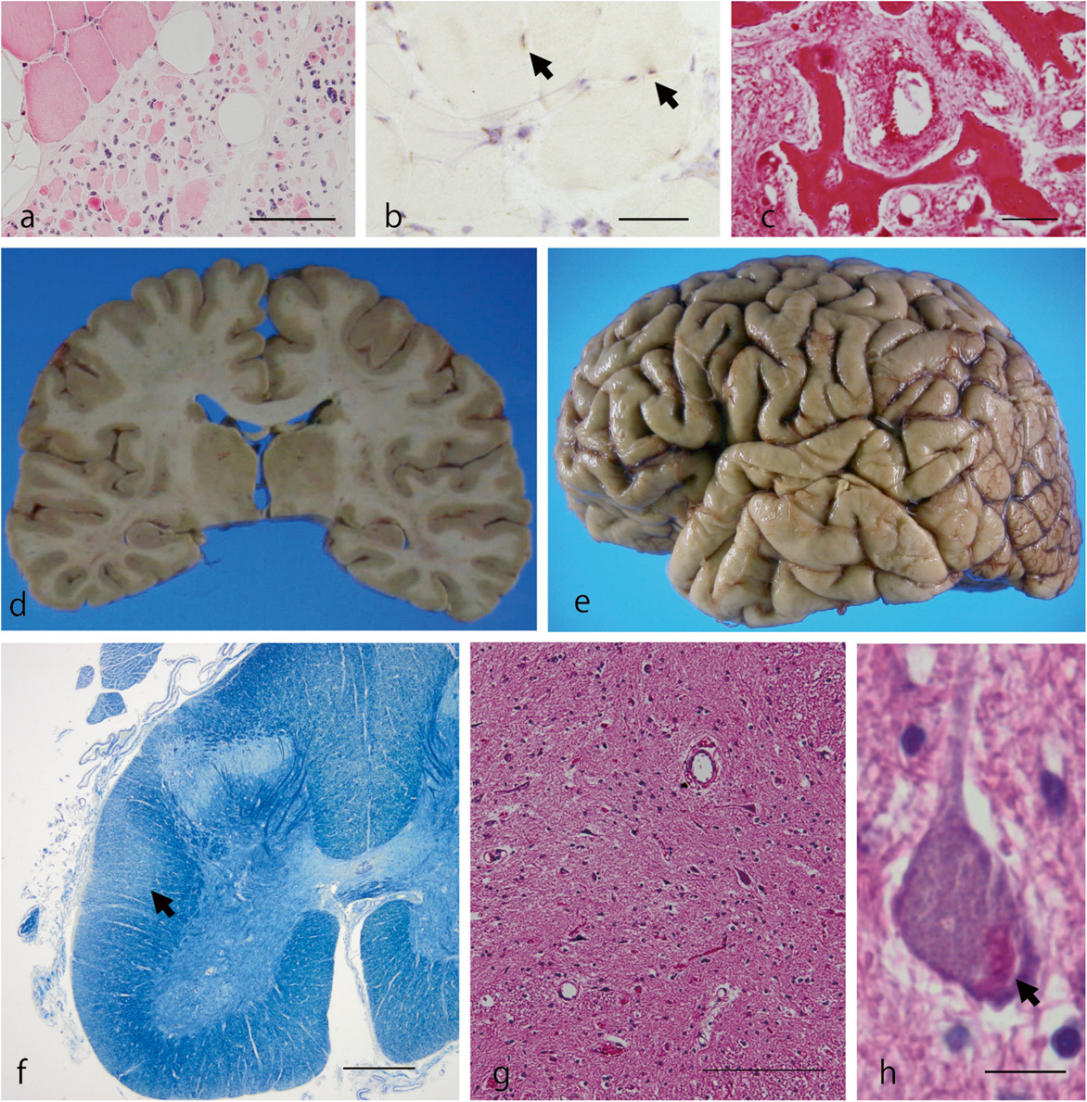
**Photographs and photomicrographs of general pathology and neuropathology of the ALS patient with the** ***VCP*** **mutation. (a,b)** In this muscle specimen from the right quadriceps muscle, large-group atrophy indicating neurogenic changes is demonstrated, whereas no evidence of IBM is observed **(a)**. The muscle nuclei are normally immunopositive with the anti-TDP-43 antibody (**b**, *arrows*), but abnormal inclusions are not detected. **(c)** H&E staining of a vertebra demonstrates irregular broad trabeculae with disorganized cement lines appearing in a mosaic pattern. **(d,e)** Macroscopically, the brain shows no apparent atrophy. **(f)** A KB-stained lumbar spinal cord reveals degeneration of the corticospinal tract (**f**, *arrow*). **(g)** A section of cervical cord stained by H&E shows neuronal loss with gliosis in the anterior horn. **(h)** Typical Bunina bodies (*arrow*) are evident in the cytoplasm of this cervical AHC. *Scale bars* = 250 μm **(a)**, 200 μm **(b)**, 50 μm **(c)**, 1 mm **(f)**, 100 μm **(g)**, and 12.5 μm **(h)**.

Frozen muscle specimens from the right quadriceps muscle at age 39 showed neurogenic changes without evidence of IBM (Figure 3a). In the immunohistochemical investigation, TDP-43 was normally detected in the muscle nuclei, which showed no abnormal inclusions (Figure 3b). Immunostaining for neither VCP nor OPTN revealed any abnormal inclusions.

At autopsy of the M158V ALS-VCP patient, pneumonia, fatty liver, and atheromatous plaques of the aorta were recognized. Microscopic examination of the vertebra revealed mixed osteoclastic-osteoblastic activity and a chaotic picture of trabecular bone (“mosaic” pattern) that was compatible with Paget disease of bone(Figure 3c).

Neuropathologically, the formalin-fixed brain weighed 1,435 g. Macroscopic examination showed no evidence of cerebral atrophy (Figure 3d, e). In H&E- and KB-stained sections, the spinal anterior horns and the corticospinal tracts showed evidence of degeneration, especially in the lumbar cord (Figure 3f). Cervical and lumbar cord showed cell loss with gliosis in the anterior horns (Figure 3g). Onuf’s nucleus and Clarke’s column were preserved. H&E-stained sections revealed the presence of Bunina bodies (Figure 3h) and spheroids in the anterior horns of the cervical and lumbar cords.

**Figure 4:**
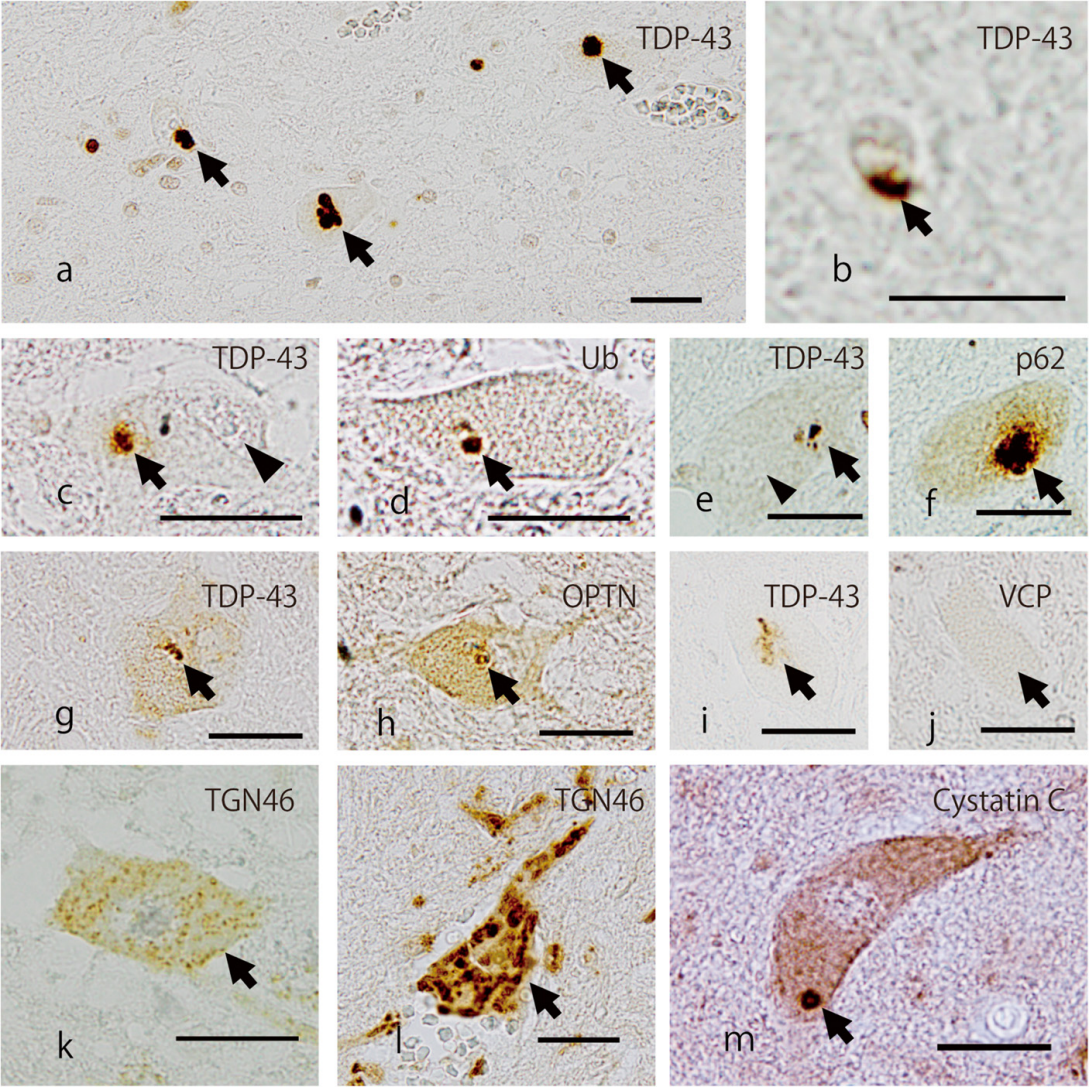
**Representative photomicrographs of the lumbar anterior horn (a-l) and the hypoglossal nucleus (m).** Intracytoplasmic inclusion bodies immunoreactive for TDP-43 are identifiable in the lumbar AHCs (**a**, *arrows*). A glial cytoplasmic inclusion (GCI) is evident with the anti-TDP-43 antibody (**b,** *arrow*). Analyses of consecutive sections indicated that TDP-43-positive inclusions in the AHCs (**c**, **e**, **g**, **i**, *arrows*) are also positive for ubiquitin (Ub; **d**, *arrow*), for p62 (**f**, *arrow*), and for OPTN (**h**, *arrow*), but devoid of immunoreactivity for VCP (**j**, *arrow*). The nucleus of the inclusion-bearing neuron lacks immunolabeling for TDP-43 (**c**, **e** *arrowhead*). Fragmentation of the Golgi apparatus is apparent in the AHCs immunostained with TGN-46, a marker protein of the Golgi, in comparison with the preserved Golgi apparatus in the non-motor neuron of the posterior horn (**l,** *arrow*). A Bunina body in the hypoglossal nucleus is immunopositive for cystatin C **(m)**. *Scale bars* = 25 μm **(a, c-m)**, 12.5 μm **(b)**.

Immunohistochemical investigation demonstrated TDP-43-positive intracytoplasmic inclusions in the AHCs (Figure 4a). GCIs positive for TDP-43 were sparsely scattered in the spinal cord (Figure 4b). The TDP-43-positive intracytoplasmic inclusions were also reactive with the anti-ubiquitin antibody (Figure 4c,d). The nucleus of these inclusion-bearing neurons was invariably immunonegative for TDP-43 (Figure 4c). Analysis of consecutive sections revealed that these TDP-43-positive inclusions were also reactive with anti-p62 antibody (Figure 4e,f) and anti-OPTN antibody (Figure 4g,h). However, TDP-43-positive inclusions (Figure 4i) were indiscernible on the VCP-immunostained sections (Figure 4j). Careful examination of 164 AHCs in 8 VCP-stained sections from the cervical, thoracic, and lumbar spinal cords revealed no immunopositive inclusions with any of the VCP antibodies. Analysis of serial sections disclosed no apparent correlation between the presence of TDP-43-positive inclusions and nuclear and cytoplasmic distribution of VCP immunoreactivity in AHCs. In the superior frontal cortex, temporal cortex, motor cortex, and hippocampus, there was no significant immunoreactivity with VCP antibody.

Immunohistochemical investigation of the Golgi apparatus revealed characteristic fragmentation of it in the AHCs (Figure 4k), whereas non-motor neurons in the posterior horn had preserved Golgi apparatuses (Figure 4l). Using the same method reported previously [39], we found that in our case 61.5% (16/26) of the AHCs from 3 distinct spinal cord segments had a fragmented Golgi apparatus. In the corticospinal tracts, immunostaining for CD68 showed infiltration of CD68-positive microglia.

**Figure 5:**
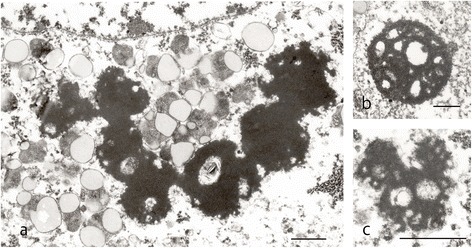
**Electron microscopy of Bunina bodies.** Three instances of Bunina bodies with irregularly shaped, amorphous, electron-dense material including vesicles and cisternae in the motor neurons of hypoglossal nuclei are depicted. *Scale bars* = 1 μm **(a-c)**.

In the hypoglossal nuclei, the motoneurons were depleted in number. Within the cytoplasm of the residual neurons Bunina bodies and TDP-43- and p62-positive inclusions were identified. The Bunina bodies were immunopositive for cystatin C (Figure 4m). Electron microscopy demonstrated Bunina bodies consisting of irregularly shaped electron-dense material containing vesicles (Figure 5a-c).

Betz cells were not apparently depleted. Hippocampal sclerosis was not found. In the primary motor, superior frontal, and temporal cortices, putamen, thalamus, cerebellum, and hippocampus, there were no neuronal nuclear or intracytoplasmic inclusions or GCIs found by any of the staining procedures, including p62 immunohistochemistry.

By amyloid β and AT8-immunohistochemistry, this case showed no senile plaque, but only a few isolated neurofibrillary tangles in the hippocampus and amygdala (CERAD 0, Braak-Braak stage II). Immunostaining for α-synuclein and FUS revealed no pathologies.

### Bioinformatics and *in vitro* studies

The effect of the newly detected missense mutation on VCP protein structure was analyzed by *in silico* analysis using different software. In Mutation Taster (http://www.mutationtaster.org), M158V was estimated to be disease causing (probability > 0.9999), and no Single Nucleotide Polymorphism (SNP) was found in the site of mutation. The known disease-causing VCP mutations R155H and R159H were also diagnosed as disease causing (probability > 0.9999) in Mutation Taster.

In SIFT (http://sift.bii.a-star.edu.sg/) the M158V mutation, as well as the known mutation R155H, was not tolerated (p = 0.00), meaning that the mutation is deleterious. On the other hand, the known mutation R159H [28,34,38] was tolerated (p = 0.07, in tolerant threshold <0.05), although the p-value was near the threshold. Polyphen-2 (http://genetics.bwh.harvard.edu/pph/) analysis showed possible damage to the structure by M158V, as indicated by a Position-Specific Independent Counts (PSIC) value of 0.896 (sensitivity: 0.82; specificity: 0.94), R155H was analyzed to be possibly damaging with a score with PSIC value 0.849 (sensitivity: 0.83; specificity: 0.93); although R159H ,which is indeed a disease-causing mutation, was predicted to be benign with a score of PSIC value 0.1 (sensitivity: 0.93; specificity: 0.85). Taken together, *in silico* analysis strongly suggested that out novel mutation M158V is pathogenic.

**Figure 6:**
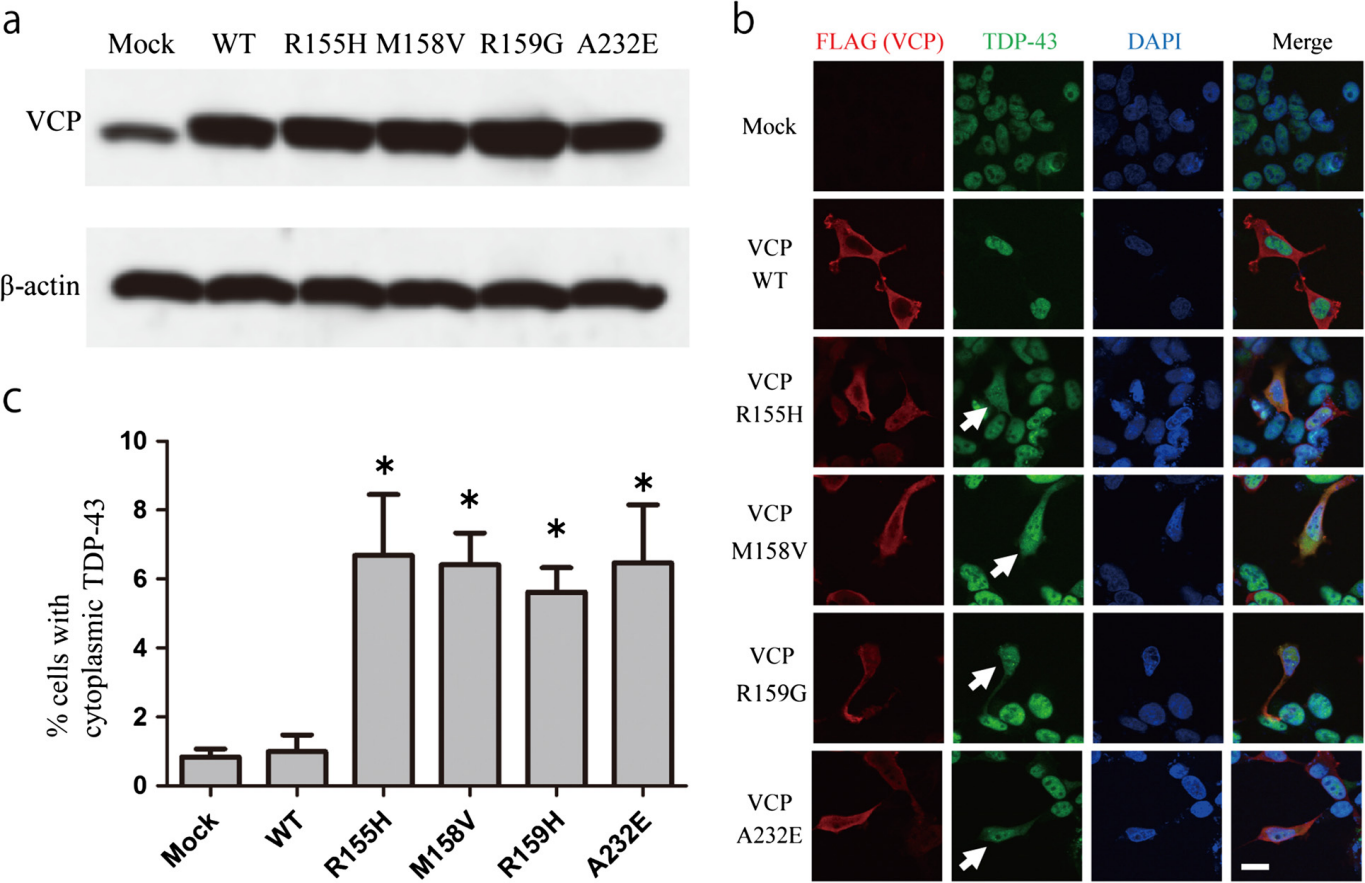
***In vitro*** **studies (SH-SY5Y cells).** Immunoblotting detects overexpression of wild-type (WT) and mutant *VCPs* in transfected SH-SY5Y cells at 48 hours after transfection **(a)**. Expression of any mutant VCP (R155H, M158V, R159H or A232E) is associated with significantly more cytoplasmic TDP-43 **(b, c)**. Values are the means from 5 trials; error bars represent standard deviation; *p < 0.05, One-way ANOVA of Bonferroni’s multiple-comparison, compared with mock and WT. The arrows in “**b**” indicate cytoplasmic TDP-43-positive cells.

To assess the functional importance of the novel mutation identified in our patient, we created mammalian expressing vectors for WT *VCP* and mutant *VCPs* (R155H, M158V, R159H, and A232E) tagged with FLAG at their N-terminus. Immunoblotting detected overexpression of WT and mutant VCP in the transfected SH-SY5Y cells (Figure 6a) and HEK293T (Additional file 1: Figure S1a), compared with the expression in the mock-transfected cells, at 48 hours after transfection. TDP-43 is a predominantly a nuclear protein, and translocation of it from the nucleus to the cytoplasm and aggregation therein are features of previously reported types of mutant *VCP-*related ALS [26,28]. Mutations in VCP were also reported to cause abnormal TDP-43 translocation in cultured cells including SH-SY5Y [40] and HEK293T [41]. So we investigated whether our novel mutation (M158V) caused the translocation of TDP-43 from the nucleus to the cytoplasm. Immunocytochemistry showed TDP-43 to be located in the nuclei of mock-transfected cells and in those transfected with WT-VCP (Figure 6b). In the mutant VCP-expressing cells, immunoreactivity of TDP-43 was often observed in the cytoplasm by 48 hours after transfection (Figure 6b). However, no evident aggregation of TDP-43 or VCP was observed in the transfected cells. Quantitative evaluation of cytoplasmic TDP-43-positive cells demonstrated that the expression of mutant VCP was significantly associated with the presence of cytoplasmic TDP-43 (Figure 6c). There was no statistical difference in the number of cells with cytoplasmic TDP-43 between the cultured cells transfected with different *VCP* mutations. In the study using HEK293T, an increase in cytoplasmic TDP-43 translocation was also observed in cells transfected with mutant VCP (Additional file 1: Figure S1b, c).

## Discussion

In the present study, we provided detailed neuropathology of an ALS case with the novel M158V *VCP* mutation, focusing on the similarity and difference to sporadic ALS. In addition, for the first time we demonstrated that the frequency of AHCs with VCP-immunoreactive cytoplasm and nucleus was increased in SALS patients as well as in this mutant case as compared with that in the controls.

Besides concomitant Paget disease of bone, the neurological manifestations and neuropathologic findings of our novel ALS-VCP patient were indistinguishable from those of the SALS patients. To date, 2 autopsied ALS-VCP cases with distinct mutations have been reported [26,28]. Clinically, both cases were diagnosed as ALS without symptoms of FTD. The initial patient was associated with an R155H *VCP* mutation. Because of the limitation of specimen availability, neuropathologic examinations of this case were confined to brainstem and spinal cord motor neurons, revealing the loss of these neurons and the presence of Bunina bodies and TDP-43-positive cytoplasmic inclusions with concomitant loss of nuclear staining in the surviving motor neurons [26]. The disease of the second autopsied ALS-VCP patient was associated with an R159H *VCP* mutation. Neuropathologic description of this case was also limited, literally describing the presence of p62-, ubiquitin-, and TDP-43-positive inclusions. Additionally, they indicated a few p62- and ubiquitin-positive inclusions in the neurons of the hippocampal granular layer and frontotemporal lobes [28]. Although neocortical inclusion pathology was not apparent in our novel ALS-VCP case, the patient exhibited otherwise similar neuropathologic findings as reported in the previous 2 cases. Furthermore, we demonstrated that in our mutant case TDP-43 was co-localized with ubiquitin, p62, and optineurin, but not with VCP, in the cytoplasmic inclusions in the AHCs. TDP-43-positive GCIs were obviously present. The lack of VCP immunoreactivity in the TDP-43-positive inclusions may imply that the M158V mutant VCP would have lost ability to associate with aggregated TDP-43. In addition, we disclosed that the Bunina bodies in our ALS-VCP patient showed immunohistochemical and ultrastructural properties indistinguishable from those observed in SALS patients. Furthermore, we identified fragmented Golgi apparatuses in our mutant case. This finding is noteworthy, because several reports have described that the Golgi apparatus is frequently fragmented in the AHCs of patients with SALS [42-44]. It is plausible that VCP dysfunction led to vulnerability of the Golgi apparatus in the present case, because VCP plays an important role in assembly of the Golgi apparatus membrane [45]. Taken together, our observations on the neuropathology of the present ALS-VCP case resembled considerably those on that of SALS, supporting the idea that VCP would underlie the pathomechanism of SALS.

Patients with FTD in IBMPFD associated with a *VCP* mutation are known to show lentiform intranuclear inclusions in the neurons of their neocortex [23-25]; however, the present case showed no such inclusions there. One possibility for this discrepancy would be the presence of modifier genes. Kimonis et al. evaluated modifier genes in a database of 231 members of 15 original families with IBMPFD and suggested a potential link between the APOE 4 genotype and FTD [10]. Alternatively, the mutation site might influence the phenotype. Recently, a study using a large data set of patients with *VCP* mutations suggested that several mutations (R155C, A232E) are correlated with a severe phenotype and reduced survival; although the precise genotypic correlation with respect to *VCP* mutation has not been elucidated yet, because some groups of mutations were very small in number [27].

The mutation site of our case (M158V) was very close to the reported sites of mutation leading to the ALS phenotype (R155H [26] and R159H [28]). In protein feature analysis with Mutation Taster, these mutations were shown to be disease causing. (Although only the R159H mutation was analyzed as moderately damaging in SIFT and Polyphen-2, this mutation in ALS and IBMPFD was reported to occur in various cases [28,34,38]) All 3 of these sites are located within the N-terminal domain, which contains ubiquitin and cofactor-binding domains (Figure 1d) [12]. The accumulation of p62 and OPTN in our case suggests that the autophagy system was defective in this ALS-VCP patient, because VCP [46-48], p62 [49], and OPTN [50] play important roles in the autophagy-dependent protein clearance system. Our results and previous reports [40,41] revealed that cells transfected with mutant *VCP* showed abnormal TDP-43 translocation. These results support the notion that neurodegeneration in *VCP* mutant cases would be attributable to dysfunction of protein clearance systems, including ubiquitin-dependent protein degradation and autophagy.

We did not detect VCP-positive inclusion bodies in our case with M158V ALS-VCP. The absence of VCP inclusions in our patient is in consistent with previous findings obtained from FTD patients with *VCP* mutations [14,23,25]. The lack of VCP-positive inclusion bodies makes a toxic gain-of-function mechanism implausible. On the other hand, since normal VCP [1] and mutant VCP [46] are reported to form heteromeric complexes, a mutant VCP could conceivably impair the formation of properly functioning hexamers, thus having a dominant-negative effect [25].

Immunohistochemical investigations on the SALS cases revealed, similar to those on the ALS-VCP patient, increased frequencies of VCP-immunoreactive structures such as neuronal cytoplasm, neuronal nuclei, and glial nuclei. These findings suggest that SALS could share its pathomechanism with ALS-VCP through dysfunctional VCP. The cytoplasm of AHCs in SALS was moderately and diffusely immunoreactive for VCP. Endoplasmic reticulum (ER) stress due to accumulation of misfolded proteins [51,52] and activated autophagy [53] in the cytoplasm have been reported to occur in SALS cases. Considering that VCP governs protein degradation processes in both ER-associated ubiquitin-dependent protein degradation and autophagy [1,4], the increase in cytoplasmic VCP in SALS may reflect a process that modifies these protein degradation systems. Neuronal and glial nuclei were more frequently immunopositive for VCP in SALS cases than in the controls. VCP-positive nuclei have also been detected in the neocortical neurons [31] and muscle cells [20] in IBMPFD cases with a VCP mutation (MSP1). On the other hand, in our study the frequency of VCP-positive neuronal nuclei in the ALS-VCP case was lower than that of these nuclei in the SALS cases, presumably because of the functional loss of the M158V mutant VCP to recognize intranuclear ubiquitinated proteins. Recently, nuclear VCP has emerged as an essential regulator of genome stability through the degradation of chromatin-associated proteins [2]. VCP is recruited at nuclear sites of damaged DNA and facilitates degradation of ubiquitinated chromatin-associated proteins to regulate DNA repair and transcription [54-56]. Notably, VCP regulates splicing pattern [3] and chromatin-associated proteins including RNA polymerase II subunit 1 (Rpb1) [56], which plays an important role in producing heterogeneous nuclear RNA (hnRNA). It is noteworthy that the alternative splicing of hnRNA is regulated by hnRNP A2/B1 and hnRNP A1 [57], which are the products of causative genes of MSP2 and MSP3 [21,22]. The dysfunction of these proteins, including VCP, hnRNP A1, and hnRNP A2/B1, could play an IBMPFD (MSP) pathogenic role through disruption of RNA metabolism and transcription processes. The increase in nuclear VCP in the ALS cases observed in the present study could be associated with the process by which VCP is recruited for intranuclear protein degradation to maintain the RNA metabolism and the transcription process. Alternatively, increased intranuclear VCP accumulation in ALS implicates loss-of-function mechanism of VCP as a pathogenesis of this disorder. Yang H et al. proposed that aggregtes of polyQ-expanded Atx3 sequester VCP into protein inclusions, and leads to neurodegeneration. It could be possible that VCP is entrapped by other intranuclear protein aggregation [58].

Our neuropathological investigations support the idea that VCP was associated with the pathomechanism of SALS and FALS with a *VCP* mutation. For determination of the pathological importance of VCP in these disorders, further studies with additional cases are warranted.

## Additional file

Additional file 1: Figure S1.
*In vitro* studies (HEK 293 T cells). Immunoblotting shows overexpression of wild-type (WT) and mutant *VCPs* of transfected HEK 293 T cells at 48 hours after transfection (a). Cells expressing any of the mutant VCPs show an increased number of expressing cytoplasmic TDP-43 compared with the cells transfected with mock or WT VCP by 48 hours after transfection (b, c). Values are the means from 3 trials; error bars represent standard deviation; *p < 0.05, One-way ANOVA of Bonferroni’s multiple-comparison, compared with mock and WT. The arrows in “c” indicate cytoplasmic TDP-43-positive cells.
