# Supplementary figures and images for: Immunoreactivity of valosin-containing protein in sporadic amyotrophic lateral sclerosis and in a case of its novel mutant
Source: Acta Neuropathol Commun. 2014 Dec 10;2:172. doi: 10.1186/s40478-014-0172-0 (PMC4297454; doi:10.1186/s40478-014-0172-0)

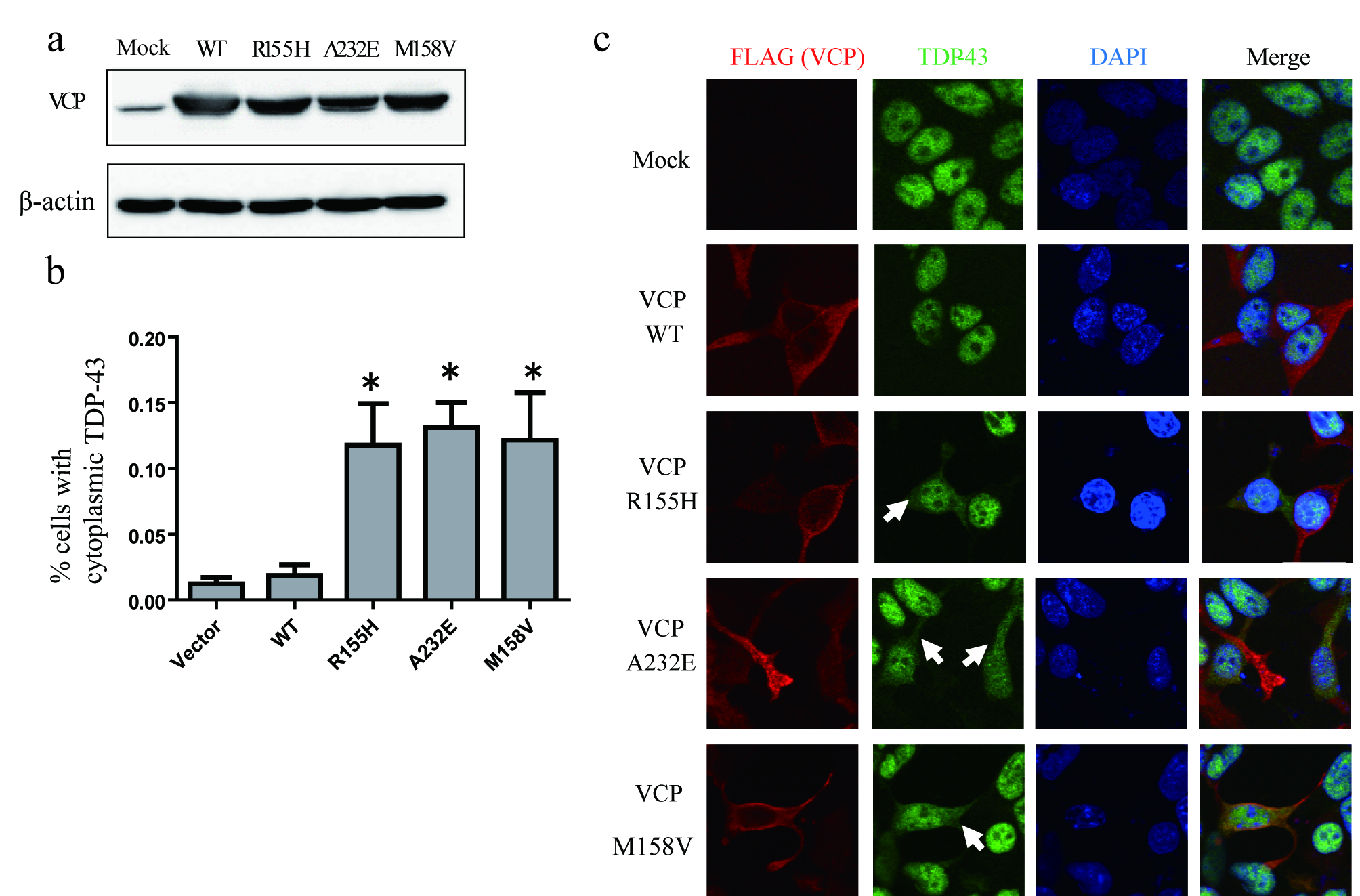

Supplement: Additional file 1: Figure S1. — In vitro studies (HEK 293 T cells). Immunoblotting shows overexpression of wild-type (WT) and mutant VCPs of transfected HEK 293 T cells at 48 hours after transfection (a). Cells expressing any of the mutant VCPs show an increased number of expressing cytoplasmic TDP-43 compared with the cells transfected with mock or WT VCP by 48 hours after transfection (b, c). Values are the means from 3 trials; error bars represent standard deviation; *p < 0.05, One-way ANOVA of Bonferroni’s multiple-comparison, compared with mock and WT. The arrows in “c” indicate cytoplasmic TDP-43-positive cells. [file 40478_2014_172_MOESM1_ESM.tiff]
